# Computer-based musical interval training program for Cochlear implant users and listeners with no known hearing loss

**DOI:** 10.3389/fnins.2022.903924

**Published:** 2022-07-27

**Authors:** Susan Rebekah Subrahmanyam Bissmeyer, Jacqueline Rose Ortiz, Helena Gan, Raymond Lee Goldsworthy

**Affiliations:** ^1^Caruso Department of Otolaryngology, Auditory Research Center, Keck School of Medicine, University of Southern California, Los Angeles, CA, United States; ^2^Department of Biomedical Engineering, Viterbi School of Engineering, University of Southern California, Los Angeles, CA, United States

**Keywords:** auditory neuroscience, cochlear implant, hearing loss, learning, music, plasticity

## Abstract

A musical interval is the difference in pitch between two sounds. The way that musical intervals are used in melodies relative to the tonal center of a key can strongly affect the emotion conveyed by the melody. The present study examines musical interval identification in people with no known hearing loss and in cochlear implant users. Pitch resolution varies widely among cochlear implant users with average resolution an order of magnitude worse than in normal hearing. The present study considers the effect of training on musical interval identification and tests for correlations between low-level psychophysics and higher-level musical abilities. The overarching hypothesis is that cochlear implant users are limited in their ability to identify musical intervals both by low-level access to frequency cues for pitch as well as higher-level mapping of the novel encoding of pitch that implants provide. Participants completed a 2-week, online interval identification training. The benchmark tests considered before and after interval identification training were pure tone detection thresholds, pure tone frequency discrimination, fundamental frequency discrimination, tonal and rhythm comparisons, and interval identification. The results indicate strong correlations between measures of pitch resolution with interval identification; however, only a small effect of training on interval identification was observed for the cochlear implant users. Discussion focuses on improving access to pitch cues for cochlear implant users and on improving auditory training for musical intervals.

## Introduction

Cochlear implants have successfully restored speech perception to people with severe hearing loss. Most cochlear implant users achieve high levels of speech recognition and spoken language skills (Shannon et al., 2004; Wilson and Dorman, 2008). However, cochlear implant users struggle to understand speech in noisy environments and many complain about the sound of music (Fetterman and Domico, 2002; Kong et al., 2004; McDermott, 2004; do Nascimento and Bevilacqua, 2005; Fu and Nogaki, 2005; Nimmons et al., 2008). Studies have shown that current cochlear implant technology is limited in its ability to convey the musical percepts of pitch and timbre (Drennan and Rubinstein, 2008; Limb and Rubinstein, 2012). This has resulted in both pitch resolution and timbre recognition being markedly diminished for cochlear implant users compared to their normal hearing peers (Gfeller et al., 2002b,^2007^; McDermott, 2004; Drennan et al., 2008; Goldsworthy et al., 2013; Limb and Roy, 2014; Goldsworthy, 2015; Luo et al., 2019). This loss of resolution and fidelity has several potential causes including limited number of implanted electrodes, electrode array placement, broad current spread, sound processing designed for speech rather than music, poor coding of timing cues for pitch, and poor neural health (Finley et al., 2008; Rebscher, 2008; Crew et al., 2012; Limb and Roy, 2014; van der Marel et al., 2014; Würfel et al., 2014; Zeng et al., 2014; Landsberger et al., 2015; Venail et al., 2015; Nogueira et al., 2016; Caldwell et al., 2017; Dhanasingh and Jolly, 2017; Mangado et al., 2018).

These technological and physiological constraints limit how music is transmitted by the implant and, consequently, limits music enjoyment for cochlear implant users. Studies have assessed adult cochlear implant user’s listening habits and music enjoyment through questionnaires (Gfeller et al., 2000; Looi and She, 2010). They found that many were dissatisfied and spent less time listening to music post-implantation. Assessment studies have also shown that cochlear implant users have more difficulty than normal hearing listeners with pitch-based perceptual tasks, including frequency discrimination and melody recognition (Gfeller et al., 2002a,^2005^, ^2007^; Penninger et al., 2013; Goldsworthy, 2015).

Melody is a fundamental aspect of music made up of a sequence of musical intervals which not only relies on the detection and direction of pitch changes, but also their magnitude. Even for those who casually listen to music, identifying the magnitude between pitches is a basic component which allows a listener to readily recognize a melody whether sung in a different register or played in a different key. If a difference in frequency cannot reliably be heard as an equivalent change in pitch, then the intended melody sounds cacophonous and out-of-tune. This has been confirmed by Luo et al. (2014) who found that cochlear implant users perceived melodies as out-of-tune more often than normal hearing listeners. Furthermore, the ability to perceive musical intervals also has implications for the emotion and tension conveyed by music. A single semitone difference between two pitches will determine the tonality of the interval (e.g., major, minor, diminished, perfect, or augmented) which, along with other important cues like timbre and tempo, will affect the listener’s emotional response to a melody (Luo and Warner, 2020; Camarena et al., 2022). The ability to reliably distinguish intervals requires listeners to have a resolution of at least a semitone (McDermott et al., 2010), and it is well established that most cochlear implant users have pitch resolution that is worse than a semitone (e.g., Pretorius and Hanekom, 2008; Goldsworthy, 2015). Without accurate perception of a musical interval, it is likely that tonality and emotion intended to be conveyed by music will be lost and this is likely a contributing factor to decreased musical enjoyment in cochlear implant users.

Musical interval labeling is an important skill for musicians and any individual who desires to participate in musical activities such as playing an instrument or singing. It is difficult to master identifying musical intervals, even in normal-hearing listeners and musicians (McDermott et al., 2010). Given the evidence discussed that suggests that musical interval perception is distorted in the context of melody perception for cochlear implant users, it is likely that cochlear implant users struggle to identify musical intervals as well. It is necessary for cochlear implant users to take steps to regain access to interval cues for musical tension and emotion. They must first undergo a period of focused aural rehabilitation to learn how the lower-level pitch cues are provided by electrical stimulation *via* their device (Gfeller, 2001), then develop the higher-level association between specific musical intervals and intent through further musical interval training (Fujioka et al., 2004).

Despite the importance of intervals to melody, there is only a small body of research investigating musical interval perception in cochlear implant users. Existing studies have shown that cochlear implant users have poor interval identification compared to their normal-hearing peers, especially above middle C. Pitch and relative intervals can be conveyed by stimulation timing (i.e., the modulation or stimulation rate) but with much variability in pitch salience and in the upper frequency that can be conveyed by stimulation rate (Pijl and Schwarz, 1995a,b; Pijl, 1997; Todd et al., 2017). Place cues for pitch (i.e., active electrodes and stimulation configuration) provide a strong sense of pitch but one that is compressed compared to normal (Stupak et al., 2021). Stupak et al. (2021) found consistent warping of intervals amongst cochlear implant users, suggesting the ability to perceive intervals is likely not linked to duration of deafness. Spitzer et al. (2021) investigated musical interval distortion in cochlear implant users who had normal hearing in their non-implanted ear (i.e., single-sided deafness). They found that the musical interval needed to create a match in the implanted ear was, on average, 1.7 times greater than the corresponding interval in the acoustic hearing ear.

Given the distorted representation of pitch and the issue of frequency compression in current cochlear implant signal processing, experience and training may be required to improve interval identification and enable access to melody through clinical devices. Interval identification is challenging for normal-hearing people and cochlear implant users alike, which makes it a demanding task for training. Moore and Amitay found that pitch training with a more difficult, or even impossible, task resulted in more robust learning (Moore and Amitay, 2007). Musical interval training in normal hearing listeners has led to improvement in both the trained and untrained tasks (Little et al., 2019). There are currently no studies investigating the effectiveness of musical interval training in cochlear implant users.

In the present study, we use an interval labeling task to evaluate subject’s ability to strengthen the association between specific musical intervals and musical intent and to consistently label intervals across an ecologically relevant musical range (i.e., the typical vocal range of humans). We note the connection between musical intervals and musical intent does not require the ability to label intervals, for example, a listener may readily associate a song in a major key as happy or bright and a song in a minor key as sad or dark (Camarena et al., 2022) without being able to label the interval pattern being used. However, given that we are interested in the restoration of a stable interval percept in cochlear implant users, we chose to use a labeling task as an important intermediary to quantify the consistency of interval labeling across musical octaves when those cochlear implant users are provided with training to the interval cues. This training task requires participants to attend to multiple musical interval presentations, associate interval magnitudes with specific labels (e.g., major third, octave), and compare presentations to intervals heard in preceding trials.

The present study has two objectives. First, to examine the performance on the trained task of interval identification and on a battery of untrained musical tasks, including frequency discrimination and tonal and rhythm comparisons before and after a two-week musical interval training program. Second, to characterize the relationship between the dimensions of music perception with low-level psychoacoustics and higher-level rhythm and tonal comparisons, interval identification, and musical sophistication. The overarching hypothesis motivating this study is that both low-level psychophysical access to pitch cues as well as higher-level labeling of intervals limits interval identification accuracy in cochlear implant users, and, to a certain extent, those with no known hearing loss. The results show that the low-level psychophysical tasks probing pitch resolution serve as predictors of higher-level measures of music perception. The results also clarify the extent that interval training improves access to the low-level and higher-level cues necessary for music perception. Discussion focuses on the importance of basic elements of pitch perception for reestablishing musical interval perception for cochlear implant users and on methods for improving training programs for musical interval identification.

## Materials and methods

### Overview

**FIGURE 1:**
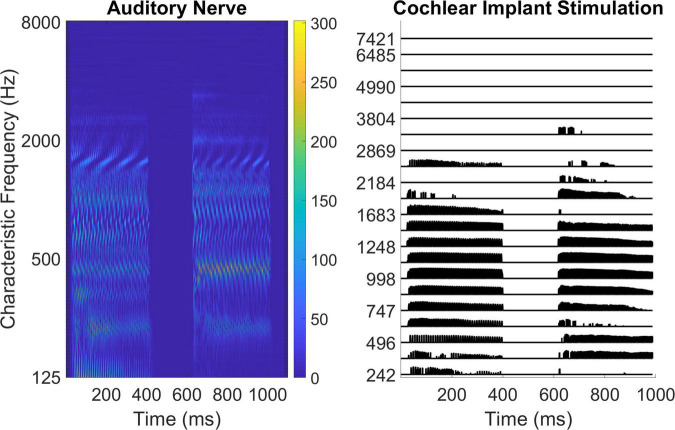
Visualizations of musical notes. The left subpanel shows auditory nerve response to musical notes for normal hearing using physiology modeling software (Zilany et al., 2014). The right subpanel shows cochlear implant stimulation patterns emulated using the Nucleus MATLAB Toolbox (Nucleus MATLAB Toolbox version 4.42, Swanson and Mauch, 2006). For both visualizations, the two notes being compared are A2 (110 Hz) and A3 (220 Hz).

Participants with no known hearing loss and cochlear implant users completed assessments before and after 2 weeks of interval training. The pre- and post-assessments included measures of pure tone detection, pure tone frequency and fundamental frequency discrimination, tonal and rhythm comparisons, and musical interval identification (the trained task) administered on the Team Hearing website coded in JavaScript. The measures of pure tone detection, pure tone frequency, and fundamental frequency discrimination used synthesized stimuli generated using JavaScript. The measures of tonal and rhythm comparisons used marimba notes rendered using Finale Version 3.5.1 software (Coda Music), and the measures of interval identification for both training and assessment used piano notes rendered using MuseScore 3 software^1^. Figure 1 shows typical normal hearing neural response patterns (left subpanel) and Cochlear Corporation cochlear implant stimulation patterns (right subpanel) for representative musical notes, highlighting the difference in frequency representation between the two groups. In the left subpanel, the 110 Hz and 220 Hz place cues can be visualized at the fundamental as well as the ascending harmonic frequencies and temporal cues can be observed with a doubling of the rate for 220 Hz. In the right subpanel, the place and temporal cues are not as clearly visualized, with the harmonic structure coarsely represented and the fundamental frequencies conveyed only through weak amplitude modulation. The place and temporal representation in cochlear implant stimulation is poor compared to the cues available for pitch perception in the normal auditory system. This representation reinforces the basis of the first part of the hypothesis, that cochlear implant users are limited in low-level psychophysical access to pitch cues. A permalink for this experiment can be found at: https://www.teamhearing.org/81, after entering the site, press the “Homework” button to enter the experiment.

### Participants

Thirteen adult cochlear implant users, with six bilaterally implanted and seven unilaterally implanted, and seven listeners with no known hearing loss took part in this experiment. All participants completed the 2-week interval training protocol. Participant ages ranged from 23 to 77 years old with an average age of 62.9 years in the cochlear implant user group and 42.3 years in listeners with no known hearing loss. Relevant subject information is provided in Table 1. Participants provided informed consent and were paid for their participation. The experimental protocol was approved by the University of Southern California Institutional Review Board.

**TABLE 1:** Subject information.

Subject	Age	Gender	Etiology	Ear tested	MSI score	Age at onset	Years implanted	CI company and processor	Implant model	Duration of deafness before implantation	Method of streaming
H1	53	M	No Known Hearing Loss	Both Together	3.61	N/A	N/A	N/A	N/A	N/A	Apple Earbuds
H2	24	F	No Known Hearing Loss	Both Together	5.89	N/A	N/A	N/A	N/A	N/A	Koss UR20 Headphones
H3	66	F	No Known Hearing Loss	Both Together	3.5	N/A	N/A	N/A	N/A	N/A	Apple Earbuds
H4	54	M	No Known Hearing Loss	Both Together	3.83	N/A	N/A	N/A	N/A	N/A	Free Field through Dell Optiplex 3080 Speakers
H5	39	M	No Known Hearing Loss	Both Together	4.33	N/A	N/A	N/A	N/A	N/A	Free Field through Panasonic TV TH-50PX80U speakers
H6	23	F	No Known Hearing Loss	Both Together	5.94	N/A	N/A	N/A	N/A	N/A	Free Field through Yamaha HS5 Powered Studio Monitor Speaker
H7	37	F	No Known Hearing Loss	Both Together	6.39	N/A	N/A	N/A	N/A	N/A	Beyer Dynamic DT 770 Pro Headphones
C2	37	F	Unknown	Both Together	4.78	15	L:9 R:13	Cochlear N7s	L:CI24RE (CA) R:CI24RE (CA)	L:5 R:1	Mini Mic2
C3	76	F	Progressive SNHL	Both Together	2.11	40	L:21 R:17	Cochlear N6s	L:CI24R (CS) R:CI24RE (CA)	L:1 R:5	Cochlear Binaural Cable
C10	46	M	Ototoxic Medicine	Left	3.28	12	33	Cochlear N6	CI22M-, United States	1	Mini Mic
C11	58	F	Sudden SNHL	Right	1.83	55	2	Advanced Bionics Naida CI Q90	HiRes Ultra 3D CI HIFocus SlimJ	1	AB Bluetooth
C13	59	M	Mumps Disease	Right	3.39	14	3	Med-El Sonnet	Sonata 2 Mi1260	42	I-loop streaming
C15	58	M	Ototoxic Medicine	Left	2	54	1	Advanced Bionics Naida	HiRes Ultra 3D CI with HiFocus Mid-Scala Electrode	1	Bluetooth/Compilot
C16	66	M	Ototoxic Medicine	Left	4.11	38	18	Cochlear N5	CI24R (CS)	5	Sony MDR-D150 Headphones
C17	74	F	Unknown	Both Together	1.78	Birth	L:20 R:15	Cochlear N6s	L:CI24R (CS) R:CI24RE (CA)	L:9 R:9	Free Field through HP Computer Speakers
C18	72	F	Measles In Utero	Both Together	2.56	Birth	L:12 R:10	Cochlear N6s	L:CI24RE (CA) R:CI512	L:1 R:1	Free Field through HP Computer Speakers
C20	67	F	Unknown	Both Together	4.11	18	L:4 R:5	L:Cochlear N6 R:Cochlear N7	L:CI522 R:CI522	L:14 R:16	Free Field through iPad Speakers
C22	65	F	Mumps Disease	Left	5.11	5	2	Cochlear N7	CI512	58	Mini Mic
C28	77	M	Unknown	Both Together	3.33	60	L:2 R:1	Med-El Rondo 3s	Synchrony 2 Mi1250	L:1 R:2	Bluetooth streaming using AudioLink
C32	63	F	Progressive SNHL	Left	6.28	20	13	Cochlear N7	CI24RE (CA)	5	Direct Bluetooth streaming from iPad

Age at time of testing and age at onset of hearing loss (when applicable) is given in years. Duration of profound hearing loss prior to implantation (when applicable) is given in years and estimated from subject interviews. SNHL, Sensorineural Hearing Loss.

### Training

All assessments and the musical interval training program were completed remotely by participants using a web application. For training, participants complete six listening exercises each day requiring approximately 20 min each day for 2 weeks. Each listening exercise included twenty trials of interval identification for which participants needed to identify 80% of the intervals correctly to proceed to the next difficulty training level. Levels were organized into thirty-six increasingly difficult levels with fewer comparisons and larger interval spacings on lower difficulty training levels.

For each trial, listeners were presented with an ascending musical interval and asked to indicate the interval that they heard. The online interface displayed two to four response buttons on screen depending on the level, with specific musical interval labels provided for selection. In total, training was provided for six different ascending melodic intervals consisting of two sequentially presented piano notes. The intervals presented and the corresponding semitone spacings between notes are listed in Table 2. Practice was provided for intervals with base notes near A2 (110 Hz), A3 (220 Hz), and A4 (440 Hz). These training levels were divided into 6 different interval groupings with 6 base note frequencies within each interval grouping. The interval groupings, described in semitone spacing between notes, were [2,12], [2,7], [7,12], [4,7,12], [2,4,7], and [1,2,3,4]. The base note frequencies within each interval grouping were (1) A2 (110 Hz) no variation, (2) A2 (110 Hz) +/- 6 semitones, (3) A3 (220 Hz) no variation, (4) A3 (220 Hz) +/- 6 semitones, (5) A4 (440 Hz) no variation, and (6) A4 (440 Hz) +/- 6 semitones. See Supplementary Table 1 for more information about the training levels. Feedback was displayed after each response on the response button selected with a green check mark for correct answers and a red “X” for wrong answers. For wrong answers, participants were given the correct answer on screen and the option to replay the interval comparison as needed.

**TABLE 2:** Interval notation with the corresponding semitone spacing between notes.

Interval	Semitone spacing
Minor 2nd	1
Major 2nd	2
Minor 3rd	3
Major 3rd	4
Perfect 5th	7
Octave	12

### Pre- and post-training assessments

Participants completed pre- and post-training assessments to characterize the effect of training on the trained task and on untrained measures of pitch discrimination and music perception. The assessments included pure tone detection, pure tone frequency discrimination, fundamental frequency discrimination, tonal and rhythm comparisons, and musical interval identification.

#### Calibration procedures

Before completing the assessments, participants completed two procedures to characterize relative loudness levels with their devices (computer, audio device, hearing device, etc.) kept how the subject would normally listen. First, participants were asked to use a method of adjustment to set a 1 kHz pure tone to subjective “soft,” “medium soft,” “medium,” and “medium loud” intensity levels in dB relative to the maximum output level of sound card without clipping. Second, pure tone detection thresholds were measured in dB relative to the maximum output level of sound card at 250, 1,000, and 4,000 Hz to provide a comparison of relative detection levels across frequencies. Stimuli were 400 ms sinusoids with 20 ms raised-cosine attack and release ramps. At the beginning of a measurement run, participants set the volume to a “soft but audible” level. The detection thresholds were then measured using a three-alternative, three-interval, forced-choice procedure in which two of the intervals contained silence and one interval contained the gain-adjusted tone. Participants were told *via* on-screen instructions to select the interval that contained the tone. The starting gain value was a threshold level as specified by the participant through method of adjustment. This value was reduced by 2 dB after correct answers and increased by 6 dB after mistakes to obtain the true detection threshold level. A run continued until three mistakes were made and the average of the last four reversals was taken as the detection threshold. This procedure converges to 75% detection accuracy (Kaernbach, 1991). Relative dynamic range could then be calculated by subtracting the detection threshold from the comfortable listening intensity level set at 1,000 Hz. The remainder of the assessments and interval training were conducted at the volume the participant set as “comfortable.”

#### Pure tone frequency discrimination

Pure tone frequency discrimination was measured for pure tones near 250, 1,000, and 4,000 Hz. Stimuli were 400 ms in duration with 20 ms raised-cosine attack and release ramps. Discrimination was measured using a two-alternative, two-interval, forced-choice procedure where the target stimulus had an adaptively higher frequency than the standard. Participants were provided with on-screen instructions to choose the sound that was “higher in pitch.” Each measurement run began with a frequency difference of 100% (an octave) between the standard and target stimuli. This frequency difference was reduced by a factor of $\sqrt[{3}]{2}$ after correct answers and increased by a factor of two after mistakes. For each trial, the precise frequency tested was roved to add perturbations which contribute to the ecological relevance of the stimulus (e.g., vocal pitch fluctuations) while avoiding both artifactual effects (e.g., sidebands outside of the filter, beating) and habituation to the base note frequency. The frequency roving was done within a quarter-octave range uniformly distributed and geometrically centered on the nominal condition frequency. Relative to the roved frequency value, the standard frequency was lowered, and the target raised by $\sqrt{1+△/100}$. The gain of the standard and target were roved by 6 dB based on a uniform distribution centered on the participant’s comfortable listening level. A run ended when the participant made four mistakes and the average of the last four reversals was taken as the discrimination threshold.

#### Fundamental frequency discrimination

Fundamental frequency discrimination was measured for fundamental frequencies near 110, 220, and 440 Hz for low pass filtered harmonic complexes. Stimuli were 400 ms in duration with 20 ms raised-cosine attack and release ramps. These fundamental frequencies were chosen as representative of the fundamental frequencies used in the interval identification assessment and training. A total of nine measurement runs were conducted consisting of three repetitions of the three fundamental frequencies. The condition order was randomized for each repetition. Harmonic complexes were constructed in the frequency domain by summing all non-zero harmonics from the fundamental to 2 kHz with a low pass filtering function. All harmonics were of equal amplitude prior to filtering. The form of the low pass filtering function was:


(1) $$g\,a\,i\,n=\left\{ \begin{array}{cc} 1 & i\,f\,f<f_{e}\\ \max\,\left(0,1-{\left(\log_{2}f-\log_{2}f_{e}\right)}^{2}\right) & o\,t\,h\,e\,r\,w\,i\,s\,e \end{array}\right.$$

where *gain* is the gain expressed as a linear multiplier applied to each harmonic component, *f* is the frequency of the component, and *f_e_* is the edge frequency of the passband, which was set as 1 kHz for the low pass filter. Note, as thus defined, the low pass filter gain is zero above 2 kHz. Fundamental frequency discrimination was measured using a two-alternative, two-interval, forced-choice procedure where the target had an adaptively higher fundamental frequency compared to the standard. The same adaptive procedure, amplitude and frequency roving, and scoring logic were used as for pure tone frequency discrimination but with adaptive control over fundamental frequency.

#### Tonal and rhythm comparisons

Participant performance on tonal and rhythm comparisons was measured using a two-alternative, two-interval, forced-choice procedure. The stimuli were the same as those generated and used by Habibi et al. (2016). In each trial, participants were presented with two 2.5 s long pre-rendered melodies rendered with marimba-like timbre, which contained 5 distinct pitches corresponding to the first 5 notes of the C major scale with fundamental frequencies ranging from 261 to 392 Hz (Habibi et al., 2016). The melodies were either the same or differed on a single note in terms of tonality or rhythm, and the listener had to choose between the on-screen options: “Same” or “Different.” The tonal and rhythm comparison procedures tested the subjects ability to identify deviations in either tonality or rhythm between pairs of unfamiliar 5-note melodies based on Western classical rules (Habibi et al., 2013, 2014, 2016). Tonal, or pitch, deviations involved the pitch change of a single note in the 5-note melody. The pitch deviations were restricted to the first 5 notes of the C major scale. Rhythm deviations involved the prolongation of a single note creating a delay in the subsequent note, the duration of which was consequently shorter so that the offset time was unchanged. The duration of each note ranged from 125 ms to 1,500 ms to create rhythmic patterns. The standard melody had no deviations in pitch or note duration. This assessment consisted of three repetitions of each set, consisting of twenty-four trials, half of which were tonal comparisons and half of which were rhythm comparisons. Performance was measured as the percentage of correct responses for each comparison domain.

#### Interval identification

Performance on musical interval identification was assessed with piano notes for three note ranges near A2, A3, and A4 (110, 220, and 440 Hz, respectively). Participants were presented with two sequentially played piano notes separated by 4, 7, or 12 semitones to represent a major 3rd, perfect 5th, or octave interval, respectively. Note, these specific test conditions corresponded to training levels 20, 22, and 24 of the training program. Responses were collected using a three-alternative forced-choice procedure where the participant had to choose between the on-screen options: “major 3rd,” perfect 5th,” or “octave.” Each measurement run consisted of twenty trials and there were three repetitions of each condition (A2, A3, A4) for a total of nine measurement runs. The musical interval chosen on any trial was randomly selected. In total, each participant completed 180 trials during the interval identification assessment and was presented with approximately 60 presentations of each of the three intervals utilized in this assessment. The base note of the comparison was roved within an octave range centered on the nominal condition note.

### The goldsmith musical sophistication index

The level of prior musical experience was measured using the Goldsmith Musical Sophistication Index Self-Report Inventory (MSI), a 39-item psychometric instrument used to quantify the amount of musical engagement, skill, and behavior of an individual (Müllensiefen et al., 2014). The questions on this assessment are grouped into five subscales: active engagement, perceptual abilities, musical training, singing abilities, and emotion. Questions under the active engagement category consider instances of deliberate interaction with music (i.e., “I listen attentively to music for *X* hours per day”). The perceptual abilities category includes questions about music listening skills (e.g., “I can tell when people sing or play out of tune”). Musical training questions inquire about individuals’ formal and non-formal music practice experiences (“I engaged in regular daily practice of a musical instrument including voice for *X* years”). Singing abilities questions inquire about individuals’ singing skills and activities (e.g., “After hearing a new song two or three times I can usually sing it by myself”). Questions under the emotion category reflect on instances of active emotional responses to music (e.g., “I sometimes choose music that can trigger shivers down my spine”). These topics together consider an individual’s holistic musical ability, including instances of formal and non-formal music training and engagement. The composite score of these subscales makes up an individual’s general musical sophistication score. All items, except those assessing musical training, are scored on a seven-point Likert scale with choices that range from *completely disagre*e to *completely agree* (Müllensiefen et al., 2014).

## Results

### Data analysis

Results from each procedure were analyzed using a mixed-effect analysis of variance. The analysis factors depended on the procedure, but all analyses included test group (cochlear implant users versus listeners with no known hearing loss) as a between-subject factor and test session (pre- versus post-training) as a within-subject factor. Planned comparisons were made between test group and test session for all assessment tasks to test whether musical interval identification training would improve the performance of the two groups on different musical tasks from pre- to post-training. Effect size was calculated using Cohen’s method (Cohen, 1992) and significance levels using multiple comparisons with Bonferroni adjustments. Comparisons between individual results across measures were performed using Pearson’s correlation coefficients.

### Pure tone detection thresholds

**FIGURE 2:**
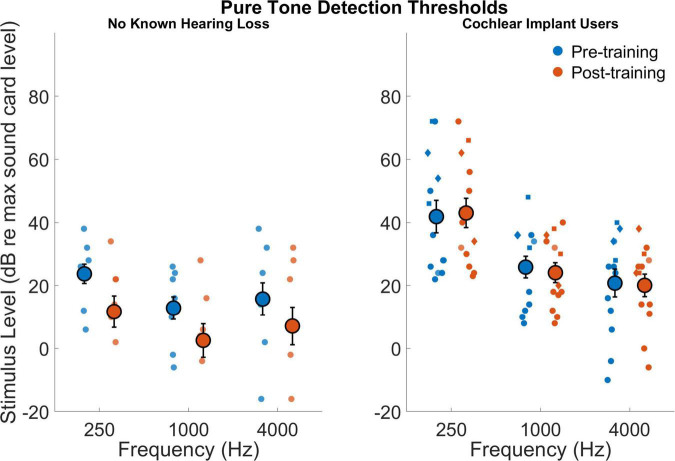
Stimulus level associated with detection threshold for 250, 1,000, and 4,000 Hz for those with no known hearing loss (left subpanel) and for cochlear implant users (right subpanel). The gain is in decibels with a gain of 100 dB corresponding to the maximum gain of the listening device. Smaller symbols indicate individual thresholds. Individual thresholds for CI users with implants from Cochlear Corporation are represented with a circle, Advanced Bionics with a diamond, and MED-EL with a square. Larger circles indicate group averages for each session with error bars indicating standard errors of the means.

Figure 2 shows the pure tone detection thresholds measured as a calibration procedure in dB relative to soundcard at 250, 1,000, and 4,000 Hz for those with no known hearing loss and for cochlear implant users. The difference in average detection thresholds between groups was significant exhibiting a large effect size (*F*_1,18_ = 10.1,*p* = 0.005,*d*_*Cohen*_ = 1.5) with cochlear implant users setting the average software volume higher (29.2=11.8) than those with no known hearing loss (12.3=10.4). Importantly, these thresholds are measured relative to the system volume that participants adjust their computers for at-home listening. These results are not indicative of absolute detection, but they do indicate that when participants adjust their computers and listening devices to be comfortable, cochlear implant users had elevated detection thresholds. It is important to note as well that relative to the self-selected comfortable listening level at 1,000 Hz, cochlear implant users had elevated detection thresholds, or a smaller relative dynamic range (*F*_1,18_ = 3,14,*p* = 0.09,*d*_*Cohen*_ = 0.7). For relative detection thresholds, the effect of frequency was significant (*F*_2,36_ = 17.3,*p* = 0.001) as was the interaction between frequency and participant group (*F*_2,36_ = 4.1,*p* = 0.024). The interaction effect is evident in the particularly elevated thresholds at 250 Hz for the cochlear implant users. The effect of session (pre- versus post-training) was not significant (*F*_1,18_ = 2.4,*p* = 0.14) nor was the interaction between session and participant group (*F*_1,18_ = 2.0,*p* = 0.17). The interaction effect of frequency and session (pre- versus post-training) was not significant (*F*_2,36_ = 0.09,*p* = 0.91) nor was the interaction for frequency, session, and participant group (*F*_2,36_ = 0.4,*p* = 0.68).

### Pure tone frequency discrimination

**FIGURE 3:**
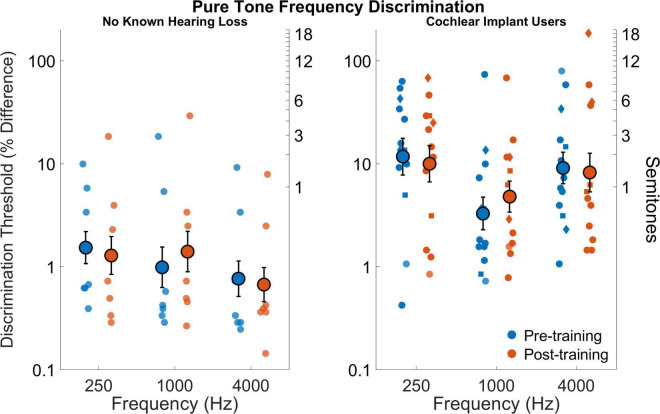
Pure tone frequency discrimination thresholds as percent difference on logarithmic scale (left y axis) and semitones (right y axis) for frequencies 250, 1,000, and 4,000 Hz for participants with no known hearing loss (left subpanel) and for cochlear implant users (right subpanel). Smaller symbols indicate individual thresholds. Individual thresholds for cochlear implant users with implants from Cochlear Corporation are represented with a circle, Advanced Bionics with a diamond, and MED-EL with a square. Larger circles indicate group averages for each session with error bars indicating standard errors of the means.

Figure 3 shows pure tone frequency discrimination for all participants before and after training. The cochlear implant users had poorer discrimination compared to those with no known hearing loss (*F*_1,18_ = 12.84,*p* = 0.002). Average discrimination thresholds across frequencies and sessions was 7.04% (or 1.18 semitones) for cochlear implant users and 1.05% (or 0.18 semitones) for those with no known hearing loss (*d*_*Cohen*_ = 1.6). There was a small effect of frequency (*F*_2,36_ = 1.95,*p* = 0.09) as well as a small effect for the interaction between frequency and participant group (*F*_2,36_ = 2.15,*p* = 0.074). The interaction effect can be seen in that discrimination improved with increasing frequency for those with no known hearing loss, but cochlear implant users had best discrimination near 1 kHz. The effect of test session was not significant (*F*_1,18_ = 0.03,*p* = 0.87) nor was the interaction between session and participant group (*F*_1,18_ = 0.006,*p* = 0.94). The interaction effect of frequency and session (pre- versus post-training) was not significant (*F*_2,36_ = 1.63,*p* = 0.21) nor was the interaction for frequency, session, and participant group (*F*_2,36_ = 0.0003,*p* = 0.99).

### Fundamental frequency discrimination

**FIGURE 4:**
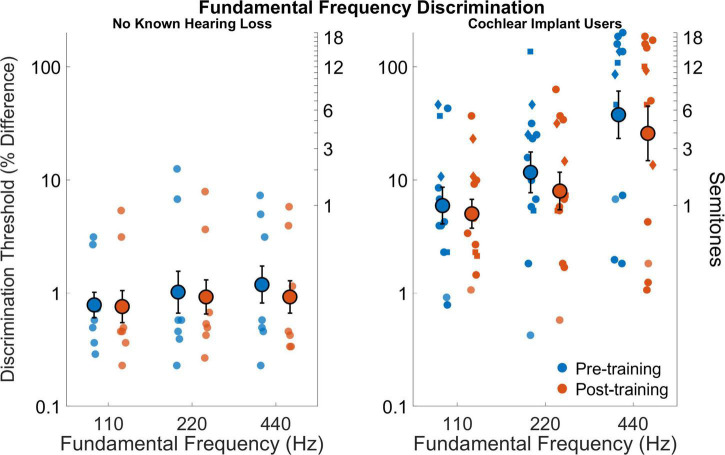
Fundamental frequency discrimination thresholds as percent difference on a logarithmic scale (left y axis) and semitones (right y axis) for fundamental frequencies 110, 220, and 440 Hz for participants with no known hearing loss (left subpanel) and for cochlear implant users (right subpanel). Smaller symbols indicate individual thresholds. Individual thresholds for cochlear implant users with implants from Cochlear Corporation are represented with a circle, Advanced Bionics with a diamond, and MED-EL with a square. Larger circles indicate group averages for each session with error bars indicating standard errors of the means.

Figure 4 shows fundamental frequency discrimination thresholds for all participants before and after training. The cochlear implant users had poorer discrimination compared to those with no known hearing loss (*F*_1,18_ = 19.3,*p* = 0.001). Average discrimination thresholds across frequencies and sessions was 11.8% (or 1.93 semitones) for cochlear implant users and 0.9% (or 0.16 semitones) for those with no known hearing loss (*d*_*Cohen*_ = 2.1). The effect of fundamental frequency was significant (*F*_2,36_ = 8.6,*p* = 0.001) as well the interaction between fundamental frequency and group (*F*_2,36_ = 4.5,*p* = 0.017). The effect of fundamental frequency is evident in that discrimination generally worsened with increasing fundamental frequency, which is more pronounced in the cochlear implant users. The effect of test session was not significant (*F*_1,18_ = 2.0,*p* = 0.18) nor was the interaction between session and participant group (*F*_1,18_ = 0.33,*p* = 0.57). Averaged across groups and conditions, the effect of training on discrimination was small but positive (*d*_*Cohen*_ = 0.13). The interaction effect of frequency and session (pre- versus post-training) was not significant (*F*_2,36_ = 0.49,*p* = 0.62) nor was the interaction for frequency, session, and participant group (*F*_2,36_ = 0.07,*p* = 0.93).

### Tonal and rhythm comparisons

**FIGURE 5:**
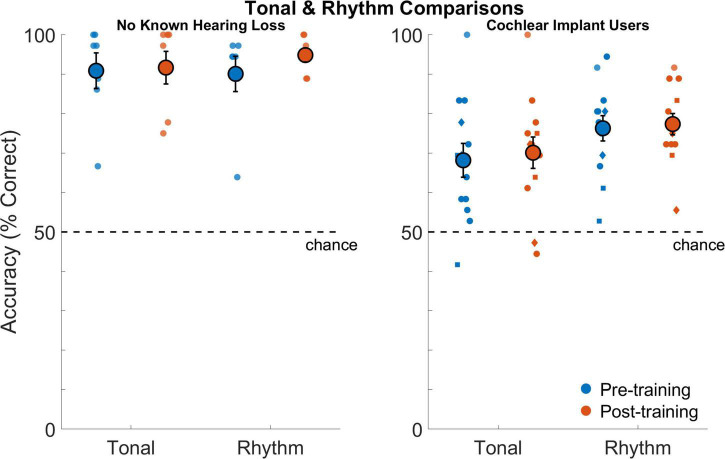
Tonal and rhythm comparisons as percentage of correct responses for listeners with no known hearing loss (left subpanel) and for cochlear implant users (right subpanel). Smaller symbols indicate individual thresholds. Individual thresholds for cochlear implant users with implants from Cochlear Corporation are represented with a circle, Advanced Bionics with a diamond, and MED-EL with a square. Larger circles indicate group averages for each session with error bars indicating standard errors of the means.

Figure 5 shows performance on tonal and rhythm comparisons for all participants before and after training. Cochlear implant users had poorer performance on tonal comparisons compared to those with no known hearing loss (*F*_1,18_ = 13.2,*p* = 0.0019). Average performance across sessions was 69.1% correct for cochlear implant users and 91.3% correct for those with no known hearing loss (*d*_*Cohen*_ = 1.85). The effect of test session was not significant (*F*_1,18_ = 0.35,*p* = 0.56) nor was the interaction between session and participant group (*F*_1,18_ = 0.01,*p* = 0.92). Neither group significantly improved on tonal comparisons across sessions.

Cochlear implant users also had poorer performance on rhythm comparisons compared to those with no known hearing loss (*F*_1,18_ = 21.5,*p* = 0.001). Average performance across sessions was 76.8% correct for cochlear implant users and 92.5% correct for those with no known hearing loss (*d*_*Cohen*_ = 1.9). The effect of test session was not significant (*F*_1,18_ = 1.75,*p* = 0.2) nor was the interaction between session and participant group (*F*_1,18_ = 1.1,*p* = 0.31). Neither group significantly improved on rhythm comparisons across sessions.

### Interval identification

**FIGURE 6:**
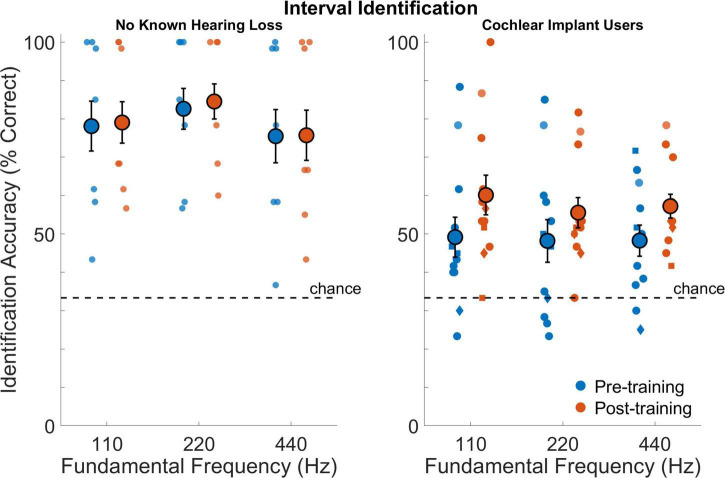
Interval identification as percentage of correct responses for participants with no known hearing loss (left subpanel) and for cochlear implant users (right subpanel) at 110, 220, and 440 Hz. Smaller symbols indicate individual thresholds. Individual thresholds for cochlear implant users with implants from Cochlear Corporation are represented with a circle, Advanced Bionics with a diamond, and MED-EL with a square. Larger circles indicate group averages for each session with error bars indicating standard errors of the means.

Figure 6 shows performance on interval identification for all participants before and after training. Cochlear implant users had poorer interval identification compared to those with no known hearing loss (*F*_1,18_ = 9.0,*p* = 0.009). Average performance across sessions was 52.4% correct for cochlear implant users and 79.2% correct for those with no known hearing loss (*d*_*Cohe*_ = 1.5). There was a no effect of frequency (*F*_2,30_ = 2.05,*p* = 0.15) but a small effect for the interaction between frequency and participant group (*F*_2,30_ = 2.94,*p* = 0.068). The effect of test session was not significant (*F*_1,18_ = 3.6,*p* = 0.076) nor was the interaction between session and participant group (*F*_1,18_ = 2.0,*p* = 0.17). Planned comparisons of the performance before and after training indicated that, on average, the cochlear implant users improved from 48.6 to 58.2% correct (*d*_*Cohen*_ = 0.63). The interaction effect of frequency and session (pre- versus post-training) was not significant (*F*_2,30_ = 0.2,*p* = 0.82) nor was the interaction for frequency, session, and participant group (*F*_2,30_ = 0.6,*p* = 0.56).

### Correlation analysis

Correlations were calculated between results from different procedures based on averages across conditions. Correlations were calculated for all participants (Table 3) and for the two participant groups separately [Table 4 (no known hearing loss) and Table 5 (cochlear implant)]. While the current measures of statistical significance for these tables are *p* = 0.05 (*), *p* = 0.01 (^**^), *p* = 0.0024 (*^x^*) and *p* = 0.001 (^***^), only the correlations with *p* = 0.0024 (*^x^*) or *p* = 0.001 (^***^) were statistically significant for the stringent Bonferroni-adjusted criteria which adjusts alpha from 0.05 to 0.05/21 or 0.0024. Considering the correlations for all participants in Table 3, all correlations, except between detection thresholds and tonal comparisons (*p* = 0.07), were significant indicating the general trend that the best performing participants were consistent across procedures. While detection thresholds were correlated with other measures, the explained variance was not as high as for the other comparisons. These correlations with detection thresholds were likely driven by group effects with cochlear implant users having elevated detection thresholds and consistently poorer performance on other measures. This notion is supported by the fact that none of the within-group correlations were significant for comparisons with detection thresholds. The low-level measures of pure tone and fundamental frequency discrimination were highly correlated with the higher-level measures of tonal and rhythm comparisons and interval identification. The strength of these correlations generally held when considering correlations within each participant group. For cochlear implant users, both pure tone and fundamental frequency discrimination were particularly well correlated to interval identification. The strong relationship between frequency discrimination and interval identification suggests that training on one of these dimensions could strengthen the other, although it is important to note that no training effects were found in this study. While fundamental frequency discrimination produced the highest correlation with interval identification, the other assessments were all significantly correlated with interval identification as well. Multiple regression analyses were calculated to determine which pairs of assessments including an interaction term provided the highest joint correlation with interval identification. The highest correlation was observed between interval identification with a multiple regression analysis of fundamental frequency discrimination and MSI scores, which produced a correlation coefficient of 0.97 when the interaction between measures was included and 0.94 when the interaction was not modeled. In general, the correlation between assessments were strongly interdependent (additional variance was not well explained by combining measures), with the most notable exception that jointly modeling MSI scores and fundamental frequency discrimination produced the largest correlation.

**TABLE 3:** Correlations between results from different procedures averaged across conditions.

	FDT	F0DT	TC	RC	II	MSI
DT	0.52*	0.55*	0.41	0.59**	0.46*	0.52*
FDT		0.94***	0.81***	0.82***	0.92***	0.72***
F0DT			0.88***	0.88***	0.93***	0.73***
TC				0.90***	0.86***	0.75***
RC					0.82***	0.70***
II						0.75***

For clarity, only the correlation magnitudes are displayed, but all comparisons were congruent in that better performance on one measure corresponded with better performance on another. Correlation coefficients and p-values associated with p-values less than 0.05 are emboldened. DT, detection thresholds; FDT, frequency discrimination thresholds; F0DT, fundamental frequency discrimination thresholds; TC, tonal comparisons; RC, rhythm comparisons; II, interval identification; MSI, musical sophistication index; p=0.05 (*), p=0.01 (**), p=0.0024 (^x^) and p=0.001 (***). Note that only the correlations with p=0.0024 (^x^) or p=0.001 (***) were statistically significant for the stringent Bonferroni-adjusted criteria which adjusts alpha from 0.05 to 0.05/21 or 0.0024.

**TABLE 4:** As for Table 3 but only including those with no known hearing loss.

	FDT	F0DT	TC	RC	II	MSI
DT	0.14	0.03	0.03	0.21	0.14	0.35
FDT		0.97***	0.88**	0.83*	0.91**	0.79*
F0DT			0.83*	0.81*	0.86*	0.69
TC				0.94^x^	0.86*	0.68
RC					0.83*	0.61
II						0.93^x^

**TABLE 5:** As for Table 3 but only including cochlear implant users.

	FDT	F0DT	TC	RC	II	MSI
DT	0.26	0.28	0.04	0.51	0.15	0.35
FDT		0.85***	0.58*	0.62*	0.84***	0.55
F0DT			0.77*^x^*	0.76**	0.94***	0.63*
TC				0.77*^x^*	0.74**	0.67*
RC					0.63*	0.61*
II						0.55

**FIGURE 7:**
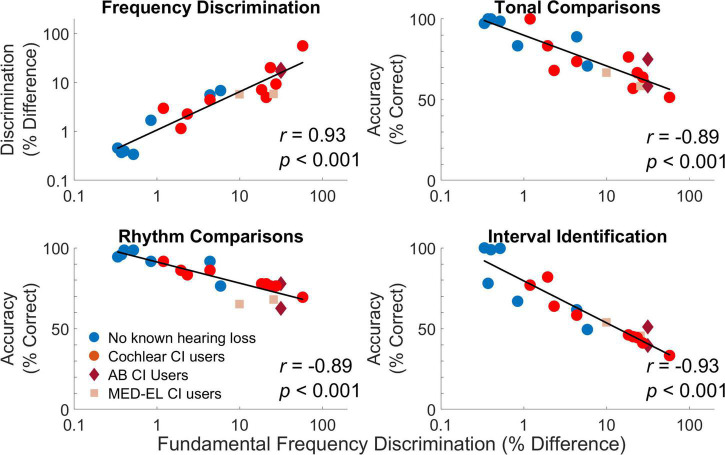
Comparisons of individual results from different procedures based on averages across conditions. For each comparison, each symbol represents the average measure for each individual participant averaged across conditions and repetitions. Individual thresholds for cochlear implant users with implants from Cochlear Corporation are represented with a circle (red), Advanced Bionics with a diamond (dark red), and MED-EL with a square (light red).

**FIGURE 8:**
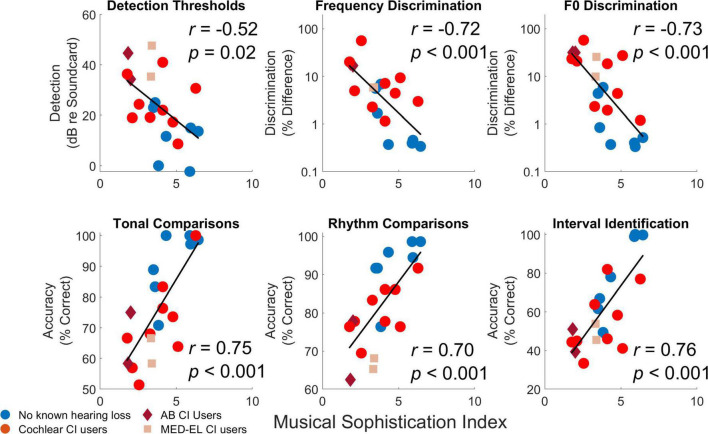
Comparisons of Musical Sophistication Index (MSI) with individual results from different procedures based on averages across conditions. For each comparison, each symbols represents the average measure for each individual participant averaged across conditions and repetitions. Individual thresholds for cochlear implant users with implants from Cochlear Corporation are represented with a circle (red), Advanced Bionics with a diamond (dark red), and MED-EL with a square (light red).

As an example of specific relationships, Figure 7 compares performance on pure tone frequency discrimination, tonal and rhythm comparisons, and interval identification with fundamental frequency discrimination. Participants who had better fundamental frequency discrimination for complex tones tended to have better performance on all other measures. As a second example of specific relationships, Figure 8 compares performance on detection thresholds, pure tone frequency discrimination, fundamental frequency discrimination, tonal comparisons, rhythm comparisons, and interval identification with MSI scores. Participants who had higher MSI scores—in particular, those with normal hearing—tended to have better performance on all other measures.

### Details of the training program

**FIGURE 9:**
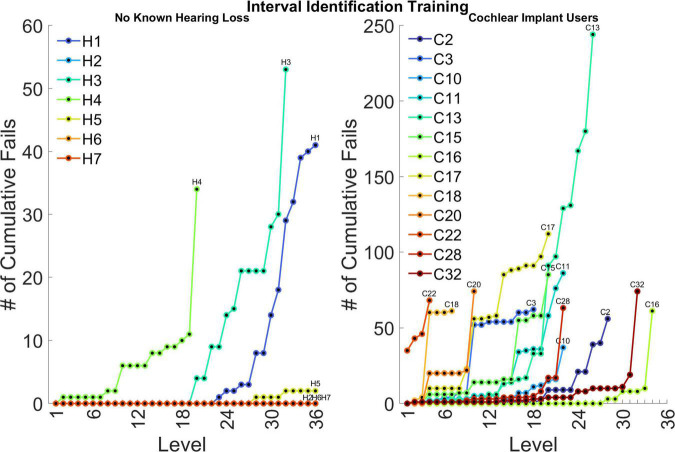
Number of cumulative failed runs across levels for individual participants. Each line ends when the participant completed 2 weeks of training or reached the final level of the training program. Note, the ordinate has a different scale for the two participant groups. The assessment conditions used for interval identification correspond to training levels of 20, 22, and 24.

Figure 9 shows the number of cumulative failed runs during training across difficulty levels for individual participants. The purpose of reporting training progress in terms of cumulative fails was to highlight which subjects had the most difficulty completing the training task at specific difficulty training levels and overall. Subjects H2, H6, and H7 had perfect performance on all difficulty training levels, so their data points overlap and only H7 is visible. Subject H5 had impressive performance as well. These four subjects were all accomplished musicians, which is reflected in their exceptional performance. The other three subjects (H1, H3, and H4) were non-musicians, with H4 struggling the most in this group. Subject C2 was a musician from a young age before getting the cochlear implant, which may have contributed to the great performance. Subjects C16 and C32 were both avid musicians who passed most difficulty training levels with ease. Subject C22 was an accomplished musician but also a bimodal listener who has not had much focused rehabilitation of the cochlear implant alone, which may have contributed to the difficulty getting past even the first level.

## Discussion

The primary aim of this study was to characterize performance on assessment tasks for cochlear implant users and listeners with no known hearing loss before and after 2 weeks of online musical interval training. Pre-training and post-training assessments measured pure tone and fundamental frequency discrimination, tonal and rhythm comparisons, and interval identification. The overarching hypothesis motivating this study is that both low-level psychophysical access to pitch cues as well as higher-level labeling of intervals limits identification accuracy in cochlear implant users, and, to a certain extent, those with no known hearing loss. Strong correlations were found between low-level measures of frequency and fundamental frequency resolution with higher-level rhythm and tonal comparisons, interval identification, and musical sophistication, thus supporting the first part of the overarching hypothesis. Furthermore, dedicated training on interval identification during this study provided cochlear implant participants opportunity to build (or rebuild) the association between interval and naming convention, along with experience with assessment tasks requiring pitch judgments.

The strength of the relationship between interval identification and frequency discrimination is well explained by separating the skills needed to perform interval identification into two components. The listener must first, hear the difference in pitch between two successive notes and second, label the magnitude of the pitch difference with the corresponding interval. Challenged in this way, participants use increasingly fine distinctions between interval magnitudes to determine the interval label. It was surprising then that a few listeners with no known hearing loss had pitch resolution at or worse than 1 semitone (note, one semitone is approximately a 6% difference in fundamental frequency). This could have been a function of age (*p* < 0.02 for correlations with PT, F0, II, and MSI), experience, unknown hearing loss, or even attention. Most cochlear implant users had pitch resolution worse than two semitones, and although age was not a factor. This poor resolution makes it difficult to form magnitude judgments, except for stark interval comparisons such as a major 2nd versus an octave. One cochlear implant user, who had pitch resolution better than a semitone, was able to correctly label 80% of intervals on the assessment task. While it was not guaranteed that the higher-level task of interval labeling would directly influence performance on lower-level psychoacoustic tasks in this brief training, given the strength of the relationship between interval identification and frequency resolution, it is possible that more extensive practice at interval labeling may transfer to simpler tasks such as pitch ranking and melodic contour identification, although this study did not find any evidence for this claim. It has also been shown that incidental listening to musical materials can improve resolution of those materials (Little et al., 2019).

The absence of significant learning in both participants groups should be taken into consideration when evaluating the effectiveness of training strategies. It has been proposed that auditory perceptual learning requires both stimulus exposure and execution of the task to be learned—provided in the current study by task practice—and a sufficient amount of practice per day (Wright, 2013). These requirements for learning must be balanced with common barriers to training paradigm success—fatigue and attrition. Studies of computer-based auditory training programs for individuals with hearing loss have varying definitions of retention and many studies do not report their compliance level (Henshaw and Ferguson, 2013). The present study aimed to make musical interval training accessible and convenient by providing an online training program that participants could use at home and by limiting training sessions to 20 min per day for 2 weeks. This is a relatively brief training protocol compared to other training programs for cochlear implant users (Looi et al., 2012). While this brief period of training likely contributed to the 100% retention rate, it may not have provided enough practice needed for learning, leading to the lack of improved performance on the trained task. Further investigation into musical interval identification training in cochlear implant users is necessary to clarify the optimal amount of daily and total training needed for learning.

An additional consideration is the difficulty of using the online interface given the age of participants in the cochlear implant user group. Technological literacy is generally lower among older populations and the mean age of the cochlear implant users was 62.9 years compared to 42.3 years for the listeners with no known hearing loss. Multiple participants reported difficulty using the online interface throughout the study. This may have made learning through the online interface difficult and training sessions may not have progressed as intended. While age did not significantly correlate with performance in the cochlear implant group, it did for the group with no known hearing loss for pure tone frequency discrimination (*p* = 0.014), fundamental frequency discrimination (*p* = 0.017), and interval identification (*p* = 0.001).

The assessment used for interval identification may also have been too difficult for the cochlear implant users. The conditions for the assessment procedure presented the participants with three types of intervals (major 3rd, perfect 5th, and octave) over three root note frequency ranges (octave ranges centered on A2, A3, and A4). These conditions correspond to training levels 20, 22, and 24 of the training program. Many participants in the cochlear implant group did not progress beyond level 22 within the 2-week training period. Therefore, one explanation for the lack of improvement in musical interval identification after training is that some participants may have only been exposed to easier levels of musical interval identification.

Our training protocol required participants to learn a difficult task in a brief amount of time. Musical intervals are a relatively abstract concept and represent the pitch ratio, a concept that is difficult to grasp without prior musical training. Given that it is well known that interval labeling is a skill that cannot be learned without dedicated musical training, a control group of participants who did not train on interval labeling was not included. It is possible that task familiarity had a small impact on participant performance that cannot be assessed without a control group, since tasks in the pre- and post-training assessments were identical. However, task familiarity is unlikely to have contributed significantly in this study given that there were no significant improvements in performance found across sessions. Furthermore, the interval labeling task was chosen due to its challenging nature, requiring participants to attend to multiple musical interval stimuli in order to progress through the difficulty training levels. Studies have suggested that an auditory task must be sufficiently difficult to result in learning since adequate amounts of attention is a requirement of learning, but there is evidence that exceptionally difficult tasks can still facilitate perceptual learning (Amitay et al., 2006; Moore and Amitay, 2007). However, the extent that task difficulty limits the higher-level labeling aspect of interval identification is poorly understood.

Musical interval identification also requires a listener to distinguish between two pitches and many listeners without prior musical training have poor resolution. McDermott et al. (2010) demonstrated that normal hearing non-musicians and even some amateur musicians had pitch interval thresholds greater than a semitone for pure and complex tone conditions. They found that interval resolution was up to 8 times worse than frequency resolution, indicating that the frequency resolution necessary to discriminate between intervals of one semitone difference in width (e.g., minor second vs major second) may need to be better than 1 semitone.

Even poorer pitch resolution is demonstrated in cochlear implant users (e.g., Pretorius and Hanekom, 2008; Goldsworthy, 2015). The ability to distinguish between two pitches is affected by the cues (temporal and place-of-excitation) provided by the processor for different stimuli (see Figure 1 for representative encoding of musical notes). Cochlear Corporation (9/13 subjects) generally discards temporal fine structure while providing temporal cues through F0 envelope modulation, MED-EL (2/13 subjects) and Advanced Bionics (2/13 subjects) attempt to encode more temporal cues through their processors, especially at lower frequencies (Arnoldner et al., 2007; Gazibegovic et al., 2010; Wouters et al., 2015). Swanson et al. (2019) unpacked the temporal and place-of-excitation cues for pure tones and harmonic complexes for the Cochlear Corporation signal processing strategy. They showed that pure tones provide only place cues to pitch, with the filter bandwidth at different frequencies having a substantial effect on the pitch resolution. For pure tones near 1,000 Hz, variation in pure tone frequency will produce variation in the relative amplitude of two neighboring filters, hence variation in currents on neighboring electrodes. For pure tones near 250 Hz, this mechanism does not work as well because the lowest filter is centered at 250 Hz, so there is no lower neighbor. For pure tones near 4,000 Hz, the filters are much wider, and if two tones are both within one filter passband, then there may be little difference in the two corresponding stimulation patterns. This may explain the general pattern of results in Figure 3 with poor resolution at both lower (<250 Hz) and higher (>4 kHz) frequencies and better resolution between 250 and 4 kHz (Pretorius and Hanekom, 2008). Our rationale for using pure tones of 250, 1,000, and 4,000 Hz is to broadly characterize spectral resolution as conveyed by place-pitch cues across the electrode array. Pure tones primarily provide place-pitch cues with the exception of strategies that attempt to provide timing cues for pure tones. While MED-EL and Advanced Bionics would attempt to provide temporal cues for 250 Hz pure tones, it does not appear to have broad effect on individual performance in Figure 3. Harmonic complexes below 220 Hz would have good temporal cues provided by amplitude modulation at the fundamental because the individual harmonics are not resolved by the ACE filter bank, between 220 and 440 would have a mixture of the two cues, and above 440 Hz would have only place cues because the individual harmonics are resolved (Swanson et al., 2019). The results of Figure 4 suggest that subjects may have been more sensitive to temporal pitch cues than place pitch cues. Interval identification was done with musical piano notes to provide the richest encoding of musical tonality (von Helmholtz, 1885; Siedenburg et al., 2019). The cues provided by these notes varied based on frequency, with trials presenting place, temporal, or a mixture of cues. Although the higher-level assessments could have been designed with stimuli to isolate a single pitch cue, as was done by Vandali et al. (2015), the present study focused on providing musical notes with the most potential cues for pitch and interval judgments, leaving the cues chosen up to each subject’s clinical signal processing strategy.

Considering the relationships between pitch resolution and music perception, this study demonstrates that pure tone frequency and fundamental frequency discrimination are both highly correlated with musical interval identification. This correlation is anticipated given that a musical interval is comprised of two different pitches. These correlations suggest that improving access to low-level cues for pure tone frequency and/or fundamental frequency perception could improve higher-level musical abilities. To improve perception of complex listening situations and musical perception for cochlear implant users, future signal processing strategies should improve access to stimulation cues that support pitch perception, whether that be through better coding of place-of-excitation cues, better coding of temporal modulation cues, or a synergy of these two (Fu and Shannon, 1999; Leigh et al., 2004; Laneau et al., 2006; Arnoldner et al., 2007; Riss et al., 2008, 2014, 2016; Stohl et al., 2008; Firszt et al., 2009; Lorens et al., 2010; Vermeire et al., 2010; Luo et al., 2012; Müller et al., 2012; Grasmeder et al., 2014; Francart et al., 2015; Rader et al., 2016; Erfanian Saeedi et al., 2017). Concomitant with better signal processing, structured aural rehabilitation programs should be designed to reintroduce cochlear implant users to the subtle stimulation cues for pitch perception. Given the correlations of low-level pitch perception with higher-level musical perception tasks, improvement in signal processing and dedicated aural rehabilitation will likely improve musical enjoyment and appreciation for cochlear implant users.

## Data availability statement

The original contributions presented in this study are included in the article/Supplementary material, further inquiries can be directed to the corresponding author.

## Ethics statement

The studies involving human participants were reviewed and approved by the University of Southern California Institutional Review Board. The patients/participants provided their written informed consent to participate in this study.

## Author contributions

JO, HG, and RG: conceptualization and visualization. SB, JO, HG, and RG: methodology, software, and validation. SB: formal analysis and data curation. SB and JO: investigation, resources, project administration, and writing—original draft preparation. SB, JO, and RG: writing—review and editing. RG: supervision and funding acquisition. All authors have read and agreed to the published version of the manuscript.

## Footnotes

1
https://musescore.org/en
