# Supplementary figures and images for: Computer-based musical interval training program for Cochlear implant users and listeners with no known hearing loss
Source: Front Neurosci. 2022 Jul 27;16:903924. doi: 10.3389/fnins.2022.903924 (PMC9363605; doi:10.3389/fnins.2022.903924)

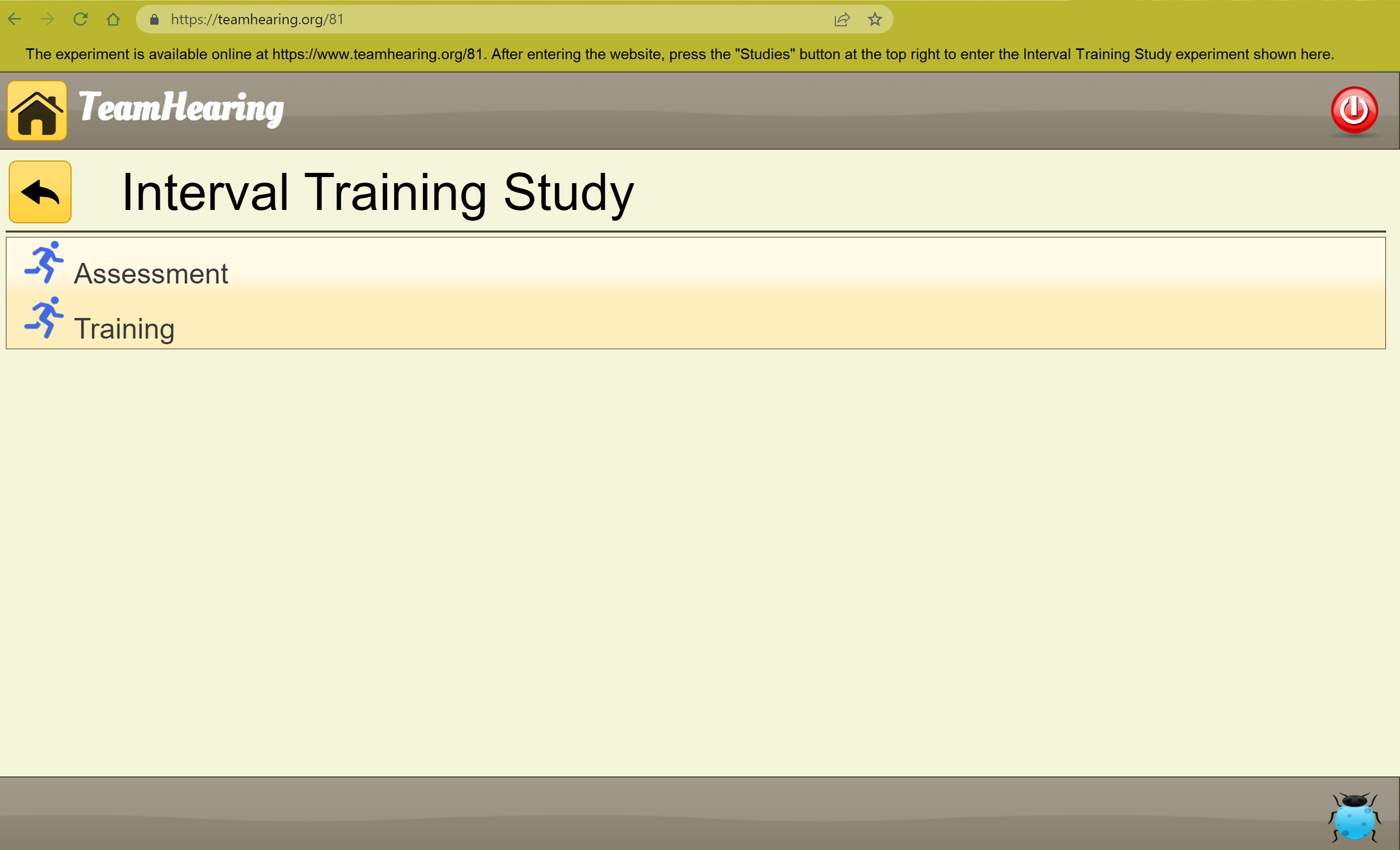

Supplement: Supplementary file 1 [file Image_1.png]
